# An avian perspective on simulating other minds

**DOI:** 10.3758/s13420-016-0230-5

**Published:** 2016-06-10

**Authors:** Nathan J. Emery, Nicola S. Clayton

**Affiliations:** 1 School of Biological & Chemical Sciences, Queen Mary University of London, London, England; 2 Biological & Experimental Psychology Group, School of Biological & Chemical Sciences, Queen Mary University of London, Mile End Road, London, E1 4NS UK; 3 Department of Psychology, University of Cambridge, Cambridge, England

**Keywords:** Theory of mind, Simulation, Corvids

## Abstract

An exciting new study on ravens by Bugnyar, Reber, and Buckner (2016) raises important questions about whether nonhuman animals are capable of simulating other minds, rather than theorizing about them.

The question of whether animals have the capacity to think about other minds remains a contentious topic in comparative cognition. Despite over 40 years of research, there is little consensus on whether any animal other than humans has a theory of mind. This research originated from a question posed by Premack and Woodruff in 1978: “Does the chimpanzee have a theory of mind?”. Despite many elegant studies using complex experimental designs, the field has generated more confusion than clarity, probably because a number of high-profile researchers have discontinued their research programs or changed their minds about their findings, or because theoretical psychologists and philosophers with little empirical training or experience testing animals in social cognitive paradigms have suggested experimental designs that they believe will fix many of the apparent problems with animal mindreading research, but that are empirically naïve. Neither is the case with Bugnyar, Reber, and Buckner (2016), two ethologists and a philosopher who have pooled their experience to design an elegant experiment that gets as close as any other to providing evidence that (some) animals may recognize (some) mental states in others.

We believe that part of the problem concerning this area of comparative cognition, more than any other, is the constraining focus on whether animals possess any aspect of human theory of mind. Although humans seamlessly make predictions about what others may be thinking, it remains unclear whether we make these predictions by scaffolding upon perceptual cues, such as another’s line of sight (theory approach), or by using our own introspection and inferences based on previous experience (simulation approach). Most research on animal mindreading has focused on the theory approach, but this has left a field littered with arguments about whether mindreading actually occurs at all in animals, and what positive evidence would look like anyway. The main argument is that animals would act similarly in response to mindreading or to behavior-reading alone, without recourse to understanding what mental states, if any, may drive another’s behavior. For example, does following another’s gaze mean that a viewer understands that the gazer is *seeing* something, or does the viewer simply compute that the gazer is oriented toward a specific object and, statistically speaking, is more likely to interact with that goal object than with another. The simulation approach does not suffer from these limitations based on behavior-reading, because it is not dependent on perceiving the links between individuals, cues, and objects. For example, I may open a box in which a toy snake springs up and scares me. If I see someone I care for approach the same box, this triggers a memory of that aversive experience, and I may try to stop my loved one from experiencing the same aversive event. I remember the state I was in while experiencing the aversive event, and would want to stop it from happening to someone I care about. But, isn’t this just my memory of something aversive, even though it didn’t happen to me? The key here is whether I would stop someone I did not like from opening the same box. My memory of the event would be just as aversive, but in this context, I might want to harm another or not care about the consequences of their actions. Introspection, in this case, would stop me from preventing another opening the box, because I would want them to experience the same aversive event that I had experienced. This has been termed *experience projection*, and we were perhaps the first to find evidence of this in a nonhuman animal, namely the Western scrub-jay (Emery & Clayton, 2001).

Briefly, jays were allowed to hide food (cache) in two different trays in two different social contexts—either in private or in the presence of another jay. Then, 3 h later, the cachers were allowed to retrieve their caches, always in private. When presented with the tray they had previously cached in in the presence of a potential observer, they consistently recached—that is, moved those caches to new places (a new tray). If presented with the tray they had cached in in private, they did not recache. We interpreted this as the birds maximizing their future returns by moving caches to places that the observing birds did not know about. However, most intriguing, and most relevant to the issue of experience projection, was the fact that we presented these two conditions to jays with experience of being thieves (having stolen caches they did not make in a previous experiment), and separately to jays with no pilfering experience. Only birds with pilfering experience performed the protective recaching behavior. We suggested that those birds alone inferred that an observer might pilfer their caches, and so implemented protective actions to prevent this possibility. As such, they may have simulated the future behavior of a potential thief on the basis of their own specific previous experience.

Unfortunately, there isn’t the space to go into arguments for why this should or should not be considered experience projection (e.g., Emery & Clayton, 2008), or most intriguing of all, why birds who had not seen a bird caching, but had stolen caches they themselves had not made, should then implement a suite of cache protection strategies when in the opposite situation (i.e., potential victims) themselves.

Bugnyar and colleagues took a different approach to the question of whether corvids simulate other minds. They adapted a paradigm first suggested by Heyes (1998) to be used with primates. Heyes suggested that chimps could wear different goggles with different colors to distinguish them—one pair transparent, the other opaque. If those experienced chimps were then confronted with other chimps wearing either the opaque or the transparent glasses (which could only be discriminated by their color), then the original chimps would behave differently toward the newcomers on the basis of their own experience of the goggles. Rather than make ravens wear goggles (and all the potential problems that could cause), Bugnyar and colleagues used peepholes to differentiate between the seeing conditions, so that birds could see into a caching arena through one peephole, but not through another. First, birds cached either when a large window was open, so that another raven could see them caching from an adjacent room, or when the window was closed, and a raven remained in the adjacent room but could not see them caching. Cachers hid the food faster and displayed fewer revisits to cache sites to improve the caches if they had been observed caching, as compared to when no observer had been present. Subjects were then given experience of the peepholes, by entering the side compartment and watching an experimenter cache in the arena through one peephole (but not through a second, blocked peephole). Finally, the subjects were moved back into the larger arena and cached when both windows were closed, but one of the peepholes in the windows was open. The clever bit was that Bugnyar and colleagues played the sounds of a raven moving in the adjacent room, from which it was unable to observe caching. Therefore, the cachers likely perceived another raven in the adjacent room who they could not see, but who had the capacity to see their caching through the peephole. In this peephole condition, the cachers behaved as if another raven was present and could see them. Bugnyar and colleagues suggested that the cachers had applied their experience of the peepholes to the potential for another bird to observe them, and so decided they should implement appropriate cache protection strategies.

Although we agree that this interpretation seems valid, there are a number of small problems. The first is that the choice of protective behaviors was completely post-hoc, and the more obvious protective behaviors did not differ between test conditions. Speed of caching or checking previous cache sites are not typical cache protection strategies. Second, and perhaps most importantly, the ravens’ decisions on where to cache did not appear to be driven by perception; that is, they did not tend to cache in locations that a potential pilferer could not see through the peephole. The peepholes themselves were quite low to the ground, with a 2-cm diameter, and it is not clear that the cacher could *not* see that no bird was present in the adjacent room. Although they behaved the same as in the observed condition, this might have been a generally protective response to a strange situation (e.g., the sounds of an unseen bird). A couple of additional peephole conditions could have been more informative, such as ones in which the peepholes were positioned higher up, and so completely out of the cacher’s sight, or both peepholes were kept open but only one had a perch next to it (so that only a bird that had experience of that perch would recognize that the perch was the only viewpoint from which a bird could spy on the caching). As songbirds, ravens should be capable of accurately pinpointing the location of a sound (such as another moving raven), so noise played from the back of the compartment could have been differentiated from noise at the location of the peephole. Perhaps in additional conditions, sounds could be played either at the back or near the peephole (the only relevant location for observing caches).

Despite these minor issues, we reiterate our earlier statement that we believe this study is a significant step forward in our understanding of whether nonhuman animals think about other minds, and that it should spark a new focus on whether animals are capable of simulating other minds.
